# Ontogenetic and spatial variability in parasite communities of white shrimp *Penaeus setiferus* (Decapoda: Penaeidae)

**DOI:** 10.1017/S0031182022001597

**Published:** 2023-03

**Authors:** Sarah R. Zuidema, Isaure de Buron, Peter R. Kingsley-Smith, Kristina M. Hill-Spanik, Natalia Fanani, Michael R. Kendrick

**Affiliations:** 1 South Carolina Department of Natural Resources, Marine Resources Research Institute, 217 Fort Johnson Road, Charleston, SC 29412, USA; 2 Department of Biology, Grice Marine Laboratory, College of Charleston, 205 Fort Johnson Road, Charleston, SC 29412, USA

**Keywords:** Apostomatida, parasite interactions, Rhabditida, shrimp health, shrimp life history, Trypanorhyncha

## Abstract

Understanding the combined effects of multi-parasite infections on their hosts is necessary for documenting parasite impacts and is particularly important for developing effective management strategies for economically important organisms. The white shrimp *Penaeus setiferus* supports important recreational and commercial fisheries along the southeastern and Gulf coasts of the United States and occupies an important ecological niche in estuarine and offshore habitats throughout these regions. The goal of this study was to identify and assess ontogenetic and spatial variation in white shrimp parasite communities and their relation to shrimp health. We used a series of trawl surveys in tidal creek and open water habitats of an estuary in the southeastern USA to collect and identify parasites of white shrimp using morphological and DNA sequencing techniques. Parasite communities in white shrimp were composed of organisms belonging to 6 classes: Conoidasida (gregarines), Oligohymenophorea (apostome and sessilid ciliates), Microsporea (meiodihaplophasids), Chromadorea (rhabditids), Cestoda (cyclophyllideans, lecanocephalideans and trypanorhynchs) and Trematoda (plagiorchiids). Parasite communities differed significantly among white shrimp life stages and localities. Furthermore, the health condition known as black gill occurred in some shrimp and was significantly related to parasite community structure. Infection metrics for the apostome ciliate *Hyalophysa lynni*, the trypanorhynch larvae *Prochristianella* sp. and the rhabditid larvae *Hysterothylacium* sp. were significantly different between shrimp exhibiting and not exhibiting black gill. These results highlight the importance of understanding parasite communities and the potential interactive effects of multiple parasite infections on shrimp health.

## Graphical abstract

**Figure:**
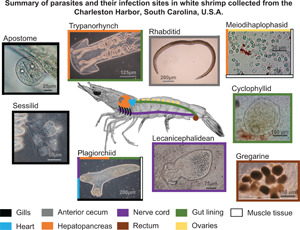

## Introduction

Single-species parasite infections have received significant attention (e.g. Lefèvre *et al*., 2008; Lafferty, 2017), but multi-parasite infections are increasingly understood as significant drivers of host health and disease outbreaks (Hellard *et al*., 2015; Clay and Rudolf, 2019). Impacts of parasite infections may include decreased host immune function, increased parasite fitness (Pedersen and Fenton, 2007; Budischak *et al*., 2015), induction of severe pathologies (Gleichsner *et al*., 2018) and altered host response to future infections (Rodríguez *et al*., 1999; Graham, 2008). Co-infections by multiple parasites can either relieve or exacerbate these impacts on host health at both the individual and population level (Reckardt and Kerth, 2009; Hellard *et al*., 2015), such that a community-level understanding of parasite infections is important for quantifying parasite impacts on host health.

Commercially important finfish and crustaceans commonly serve as hosts for a diversity of parasites in freshwater and marine environments and wherever possible their presence and impacts should be incorporated into management approaches (Marcogliese, 2004; Byers, 2021). For example, over the course of their life cycle, white shrimp, *Penaeus setiferus* (L., 1767) (Decapoda: Penaeidae) serve as hosts to a variety of parasites, including but not limited to, platyhelminthes, nematodes, ciliates and microsporidians (e.g. Overstreet, 1978; Domínguez-Machín *et al*., 2011; Del Río-Rodríguez *et al*., 2013). Penaeid shrimp are one of the most important fisheries and aquaculture industries worldwide (FAO, 2018). Specifically, adult white shrimp support important commercial and recreational fisheries along the coasts of the southeastern USA (Gillet, 2008) and the Gulf of Mexico (Muncy, 1984; NMFS, 2020), including $13.7 million annually from the Georgia Bight in the western Atlantic (Kendrick *et al*., 2021). Additionally, white shrimp provide a food source for many recreationally and ecologically important finfish, including red drum, *Sciaenops ocellatus* (see Scharf and Schlight, 2000) and spotted seatrout, *Cynoscion nebulosus* (see Fujiwara *et al*., 2016). As such, white shrimp comprise an integral part of the estuarine ecosystem and economy and monitoring population health is necessary to ensure sustainable management.

Commercial landings of white shrimp in parts of the Georgia Bight have declined during the last 2 decades (Kendrick *et al*., 2021), coinciding with numerous biotic and abiotic changes in the estuarine waters of the southeastern USA (e.g. Hollebone, 2006; Shearman and Lentz, 2010). Among these biotic changes is the increased prevalence of a condition known as black gill (Fowler *et al*., 2018; Swinford and Anderson, 2021; Tuckey *et al*., 2021). This condition refers to the melanization of gill tissues that occurs as part of an immune response against irritants or pathogens (Vaseeharan and Ramasamy, 2003; Johnson *et al*., 2011; Burnett and Burnett, 2015; Karthikeyan *et al*., 2015; Frischer *et al*., 2022). Black gill in wild-caught penaeid shrimp has been associated with the presence of the apostome ciliate *Hyalophysa lynni* (see Landers *et al*., 2020), but also occurs in response to heavy metals (Ghate, 1984), bacteria (Vaseeharan and Ramasamy, 2003) and fungi (Karthikeyan *et al*., 2015).

Parasites of white shrimp in the Georgia Bight, including South Carolina (SC) have not been intensively surveyed, and their impacts on white shrimp health at the individual and population level, and consequently on commercial landings, warrant investigation. The objectives of this study were to: (1) identify parasite community composition of white shrimp in the Charleston Harbor, SC; (2) document ontogenetic and spatial variability in the parasite communities of white shrimp; and (3) assess the relationships between parasites and black gill in white shrimp.

## Material and methods

### Collection of post-larval white shrimp

**Fig. 1.**
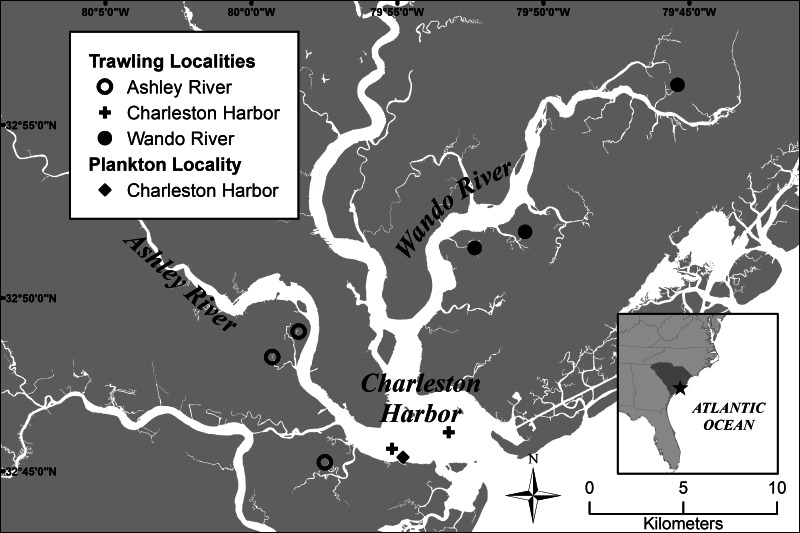
Map of sampling localities in the greater Charleston Harbor, South Carolina, USA: Wando River, Ashley River and Charleston Harbor. Credit: Gary Sundin, South Carolina Department of Natural Resources (SCDNR).

Zooplankton sampling was conducted at the surface during nocturnal flood tides that coincided with neap tides to correspond with the timing of shrimp ingress (DeLancey *et al*., 1994; Wenner *et al*., 2005). Collections occurred between 6 June 2018 and 1 September 2018 and consisted of 18 sampling events. Two 0.75 m diameter plankton nets (500 *μ*m mesh) were deployed for 30–75 min (depending on changes in tidal flow or the timing of the flood tide with respect to darkness) from a floating dock in Charleston Harbor, SC, USA (Fig. 1). Plankton samples were washed with seawater using a 500 *μ*m mesh sieve and transported in an aerated container of seawater to the laboratory. Within 24 h, post-larval shrimp were isolated using a dissecting microscope and identified as penaeid following the key of Johnson and Allen (2005). These specimens were presumed to be white shrimp based on the timing of ingress, which largely does not overlap with any other penaeid species in SC (DeLancey *et al*., 1994). Specimens were flattened between 2 slides and examined whole under a compound microscope to detect parasites.

### Collection of juvenile, subadult and adult white shrimp

Juvenile, subadult and adult white shrimp were collected within the greater Charleston Harbor watershed at each of 3 localities (Fig. 1); tidal creek localities in the Ashley River watershed (*n* = 3), tidal creek localities in the Wando River watershed (*n* = 3) and open water localities in Charleston Harbor (*n* = 2). Specimens were collected from tidal creek localities monthly from June to September 2018 (*n* = 15 per site) and following their egress from tidal creeks in late-summer, from open water localities monthly (*n* = 23 per site) from August to November 2018. Collections from tidal creek localities were made using a 3.05 m otter trawl (0.95 cm stretch mesh net) towed for 5 min at ~2 knots. Collections from open water localities were made using a 6.2 m otter trawl (2.5 cm stretch mesh net) for 15 min at ~2 knots. Bottom water temperature (°C) and salinity (psu) were recorded at each site on each collection date using a handheld YSI Pro2030. Shrimp were measured from the tip of the rostrum to the tip of the telson, and size classes were defined according to Whitaker and Kingsley-Smith (2014) as follows: juvenile (⩽114 mm length), subadult (115–126 mm length) and adult (⩾127 mm length).

### Identification of parasite community composition

Specimens were brought back on ice to the laboratory and examined for parasites immediately, or stored at –20°C for later processing. To collect parasites, individual shrimp were submerged in a dish of deionized water and, using a dissecting microscope, the carapace was removed and the organs excised from the cephalothoracic cavity. The hepatopancreas and feeding palps were deconstructed to isolate parasites, while the nerve cord was resected from the full length of the shrimp, flattened between 2 slides and examined with a compound microscope under 100× total magnification. The anterior caecum was resected from the digestive tract and opened along with the stomach, intestine and hindgut to examine their contents. A ~1–2 mm^2^ squash of the abdominal muscle was performed between 2 slides and examined at 100× total magnification. Smears were performed when gonads or muscle were infected and stained with Giemsa. Parasite specimens were fixed in 95% ethanol for molecular analysis.

As morphological identification of larval parasites can be challenging, morphotypes of parasite groups were first developed using morphological characteristics and infection sites (Overstreet, 1973, 1978; Deardorff and Overstreet, 1981; Palm, 2004; Sokolova *et al*., 2015). These morphotypes were screened using genetic sequencing for further identification. Subsamples of each morphotype group (except for the sessilid ciliates, which were not preserved) were processed for molecular analyses. DNA extractions (*n* = 26) were performed using a DNeasy Blood and Tissue kit (Qiagen, Valencia, CA, USA) following manufacturer protocols, except that elution volumes were reduced to 40 *μ*L. For each taxonomic group, primers were chosen that would amplify a gene region most likely to provide species-level identifications (Table 1).

**Table 1.** Primers used for amplification and sequencing of white shrimp parasites

Phylum	Gene region	Primer name	Primer orientation	Primer sequence (5′–3′)	Primer reference
Ciliophora	SSU	18SF-ciliate-458	+	AGCAGGCGCGHAAATTRCCCAATCY	Frischer *et al*. (2017)
		18SR-ciliate-1260	−	CCGTGTTGAGTCAAATTAAGCCG	Frischer *et al*. (2017)
Microsporidia	SSU	ss18f	+	CACCAGGTTGATTCTGCC	Ghosh and Weiss (2009)
		ss1482r	−	GGTTACCTTGTTACGACTT	Ghosh and Weiss (2009)
Nematoda	cox2	211F	+	TTTTCTAGTTATATAGATTGRTTTYAT	Nadler and Hudspeth (2000)
		210R	−	CACCAACTCTTAAAATTATC	Nadler and Hudspeth (2000)
Platyhelminthes	LSU	LSU5	+	TAGGTCGACCCGCTGAAYTTAAGCA	Jensen and Bullard (2010)
		1200R	−	GCATAGTTCACCATCTTTCGG	Jensen and Bullard (2010)

The gene region used was based on the most informative marker for species-level identification: large subunit ribosomal RNA (LSU rRNA) gene for platyhelminthes, partial small subunit (SSU) rRNA gene for ciliates and microsporidians, and mitochondrial cytochrome *c* oxidase II (cox2) for nematodes.

For primer orientation, +, sense; −, antisense.

For platyhelminthes, a 25 *μ*L total reaction contained 1× Promega GoTaq^®^ Flexi PCR Buffer (Madison, WI, USA), 0.1× Invitrogen *Redi*load™ loading buffer (Thermo Fisher Scientific, Waltham, MA, USA), 1.5 mm MgCl_2_, 0.2 mm dNTPs, each primer at 0.4 *μ*m, 0.05 U *μ*L^−1^ Promega GoTaq^®^ DNA polymerase and 1 *μ*L template DNA. Cycling was performed as described by Jensen and Bullard (2010), with one alteration in that cycling began with a 5 min denaturation at 95 °C rather than at 94 °C. For ciliates, PCR reagents and concentrations were the same as above except 0.5 *μ*m of each primer was used. Cycling was performed as described by Guo *et al*. (2012). For nematodes, a 25 *μ*L total reaction contained 1× PCR Buffer (Thermo Fisher Scientific), 0.1× Invitrogen *Redi*load™ loading buffer (Thermo Fisher Scientific), 2.5 mm MgCl_2_, 0.2 mm dNTPs, each primer at 0.2 *μ*m, 0.2 *μ*m Invitrogen Platinum™ *Taq* DNA polymerase and 1 *μ*L template DNA. Cycling was performed as follows: 5 min at 96 °C then 40 cycles at 96 °C for 40 s, 45 °C for 40 s and 72 °C for 40 s, then 72 °C for 5 min. For microsporidians, PCR reagents and concentrations were the same as for the platyhelminthes PCR (described above), except that 1 *μ*m of each primer was used in a 20 *μ*L total reaction volume. Cycling performed was as follows: 4 min at 94 °C then 35 cycles at 94 °C for 30 s, 55 °C for 30 s and 72 °C for 2 min and then at 72 °C for 5 min. For gregarines, several small subunit ribosomal RNA gene primers (Leander *et al*., 2003; Rueckert and Leander, 2009; Diakin *et al*., 2016) were tested but did not result in amplification.

All PCR products were electrophoresed on 1% agarose gels (100 V, 30 min) stained with GelRed^®^ (Biotium, Inc., Hayward, CA, USA), and visualized under UV light. Products were cleaned using ExoSAP-IT™ (Affymetrix, Santa Clara, CA, USA) following manufacturer protocols. Cleaned products were sent to Eurofins MWG Operon LLC (Louisville, KY, USA) for direct, bi-directional sequencing using the primers shown in Table 1. Complementary sequences were compared to one another and to their chromatograms using Sequencher^®^ version 5.4 (Gene Codes, Ann Arbor, MI, USA). Resulting sequences were compared against the NCBI GenBank database using the Basic Local Alignment Search Tool (BLAST; Altschul *et al*., 1990). A 99% sequence similarity threshold was used for species-level identifications for all taxa; assignments to genus level were based on percent similarity and the BLAST taxonomy report.

Parasite prevalence, intensities and densities were defined according to Bush *et al*. (1997). Mean intensities could not be determined for the cyclophyllidean, the ciliates (apostome and sessilid), the gregarine or the microsporidian (Table 2). Prevalence only was recorded for the cyclophyllidean, gregarine and microsporidian. For the ciliates, relative abundance was calculated for the apostome and estimated for the sessilid (see below). Mean intensities and relative abundances are expressed as ± standard error (s.e.).

**Table 2.** Parasite taxa identifications, BLAST results and quantitative factors used in analyses. N/A = sequencing was unsuccessful or not done. P = prevalence. Only species with intensity (I) or relative abundance (RA) were included in NMDS and associated analyses.

				BLAST results			
Phylum	Class	Order	Specimen ID	Organism name	% ID	Accession No.	Quantitative factors
Apicomplexa	Conoidasida	Gregarinasina^a^	Gregarine	N/A	N/A	N/A	P
Ciliophora	Oligohymenophorea	Apostomatida	*Hyalophysa lynni*	*Hyalophysa lynni*^b^	100	KX906567	P/RA
Ciliophora	Oligohymenophorea	Sessilida	*Zoothamnium* sp.	N/A	N/A	N/A	P/RA
Microsporidia	Microsporea	Meiodihaplophasida	*Agmasoma penaei*	*Agmasoma penaei*	100	KF549987	P
Nematoda	Chromadorea	Rhabditida	*Hysterothylacium* sp.	*Hysterothylacium reliquens*	96–97	AF179916	P/I
Platyhelminthes	Cestoda	Cyclophyllidea	*Parorchites* sp.	*Parorchites zederi*	96	KP893423	P
Platyhelminthes	Cestoda	Lecanicephalidea	*Polypocephalus* sp.	*Polypocephalus* sp.	100	GQ470200	P/I
Platyhelminthes	Cestoda	Trypanorhyncha	*Kotorella pronosoma*^c^	*Kotorella pronosoma*	100	MK558802	N/A
Platyhelminthes	Cestoda	Trypanorhyncha	*Prochristianella* sp.	*Prochristianella* sp.	98	DQ642783	P/I
Platyhelminthes	Trematoda	Plagiorchiida	*Opecoeloides fimbriatus*	*Opecoeloides fimbriatus*	100	KJ0011211	P/I

a Gregarinasina is a subclass of Apicomplexa, but specimens could not be identified past this level of classification, and are consequently referenced only as ‘gregarines’.

b As of this publication, the GenBank record had not been updated to reflect the findings of Landers *et al*. (2020) who associated this GenBank accession number with *Hyalophysa lynni*. Specimen ID agrees with organism name where BLAST results returned 99% similarity or higher, otherwise only genus level was confirmed.

c A single specimen was identified so it was included as part of the trypanorhynchs for the purpose of analyses.

### Macroscopic assessment of black gill condition

A black gill score was determined macroscopically for individual shrimp by evaluating the darkest portion of gills in each specimen in the field using a colour standard scale comprised of increasingly pigmented paint swatches as follows based on the hexadecimal colour code: 1 = #DFD3C3, 2 = #DACAB2, 3 = #CCB79B, 4 = #C0A98B, 5 = #AB9579, 6 = #947F65, 7 = #6E543C, 8 = #5A4A3D, 9 = #564536 and 10 = #48423C. A black gill score from 1 (no melanization) to 10 (darkest melanization) was recorded for each specimen, with scores >5 categorizing individuals as shrimp with black gill and scores of ⩽5 categorizing individuals as shrimp without black gill.

### Assessment of infection by gill parasites

To examine gill parasites (i.e., the sessilid and apostome ciliates), the 4 most posterior gill filaments of the haphazardly chosen left or right side were removed from each juvenile, subadult and adult shrimp (*n* = 543) and preserved in 3–5% formalin or frozen in deionized water until later examination (Martin *et al*., 2000). Wet mounts of the gill filaments were observed using light microscopy at 200× total magnification. Gill filaments were subdivided into 3 non-overlapping fields of view (FOVs) each of 0.785 mm^2^. Smaller gills typically allowed for only 1 or 2 FOVs per filament. The number of trophonts of the apostome ciliate in each FOV was recorded and relative abundance calculated as the number of apostome ciliates per mm^2^ of gill tissue. For sessilid ciliates, relative abundance was estimated according to the number of FOVs showing stalks of these parasites; this value was corrected for the number of FOVs available in an individual shrimp.

### Statistical analyses

Parasite prevalence, intensities and abundances were used for statistical analyses, which were performed in R version 4.0.3 (R Core Team, 2019) (Table 2). As only 1 individual of the trypanorhynch *Kotorella pronosoma* (Stossich, 1901) was observed, it was combined with the other trypanorhynch (*Prochristianella* sp.) in the analyses. Due to low parasite infections in white shrimp post-larvae, statistical analyses were restricted to juvenile, subadult and adult life stages. When testing how individual parasites relate to the patterns of black gill prevalence, analyses were restricted to collections made from the open water localities during the black gill season (August through October).

To assess variability among parasite communities across ontogenetic and spatial factors (i.e., localities), parasite abundances were first averaged for each trawl and then standardized using the Wisconsin double standardization tool, which standardizes species by the maximum value and then standardizes localities by totals, using the ‘vegan’ package (Oksanen *et al*., 2019). Bray–Curtis distance matrices of parasite abundances were then developed and used to create non-metric multidimensional scaling (NMDS) plots with 95% data ellipses to examine shrimp parasite communities among localities. Water temperature (°C), salinity (psu), shrimp length and black gill score were tested for correlation with multivariate distances using permutation tests with 999 iterations and visualized as vectors using the ‘envfit’ function in the ‘vegan’ package (Oksanen *et al*., 2019). Parasite taxa names overlaid onto the ordination plot illustrate where taxa correspond to one another within the ordination space. Permutational analyses of variance (PERMANOVA) and corresponding pairwise *post hoc* tests using the ‘RVAideMemoire’ package (Hervé, 2021) were also conducted to examine specific differences among parasite communities of shrimp groups (i.e., juvenile *vs* subadult *vs* adult and Ashley River *vs* Wando River *vs* open water Charleston Harbor localities).

Indicator species analyses were performed using multi-level pattern analysis (‘multipatt’ function within the ‘indicspecies’ package; de Cáceres and Legendre, 2009) to test for parasite taxa that were significantly associated with white shrimp life stage or locality. Indicator values (IV), i.e., the proportion of parasite *x* occurring in group A multiplied by the proportion of individuals in group A that contain parasite *x* (Dufrene and Legendre, 1997), were also calculated to determine how strongly a parasite grouped with a particular locality or host life stage.

Species-specific mixed-effects logistic regression analyses (‘glmer’ function within the ‘lme4’ package; Bates *et al*., 2015), with collection event included as a random effect to account for the lack of independence within sampling events, were used to assess how parasite presence related to white shrimp life stage and collection locality. Model significance was assessed using a likelihood ratio test followed by Tukey's *post hoc* analyses to identify pairwise differences performed using the ‘multcomp’ package (Hothorn *et al*., 2008). The relationship between the apostome ciliates and black gill from open water localities in fall months was tested using a binomial test and a mixed-effects segmented regression with collection event included as a random effect. Differences in parasite prevalence and intensity/relative abundance between shrimp with black gill and those without black gill were examined using mixed-effects approaches in the ‘lme4’ package (Bates *et al*., 2015) followed by Tukey's comparisons of estimated marginal means in the ‘emmeans’ package (Lenth, 2022). These models were restricted to collections in open water habitats in fall months and for quantifiable parasites found in the Harbor during these months (i.e., apostomes, rhabditids, lecanicephalideans, trypanorhynchs and plagiorchiids). For these models, random factors of individual shrimp, collection month, sampling event, date and sampling event nested within date were included if they enhanced model performance by not contributing to model overfitting.

## Results

### Parasite community composition

**Fig. 2.**
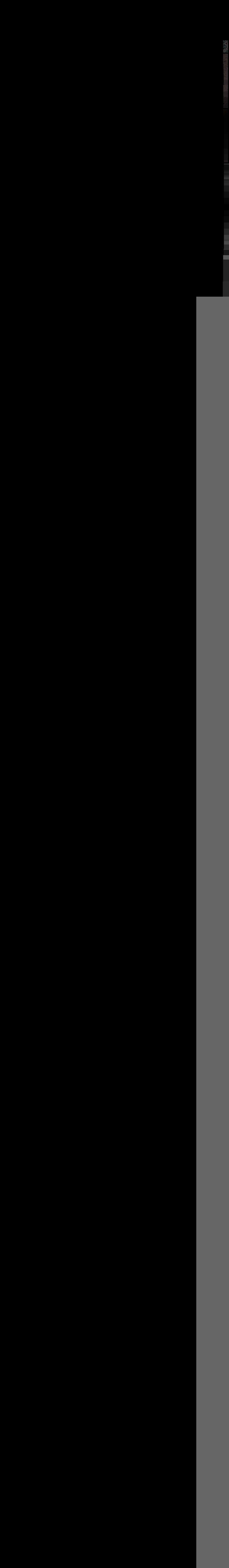
Parasites of the white shrimp, *Penaeus setiferus* observed in the Charleston Harbor watershed, South Carolina, USA. (A) Excysted metacercaria of the plagiorchiid *Opecoeloides fimbriatus*, scale bar 150 *μ*m. (B) Juvenile of the rhabditid *Hysterothylacium reliquens*, scale bar 200 *μ*m. (C) Microsporidian meiodihaplophasid *Agmasoma penaei*, scale bar 25 *μ*m. (D) Sessilid *Zoothamnium* sp., scale bar 75 *μ*m. (E) Cyclophyllidean *Parorchites zederi*, scale bar 85 *μ*m. (F) Lecanicephalidean *Polypocephalus* sp., scale bar 75 *μ*m. (G) Scolex of plerocercus of the trypanorhynch *Prochristianella* sp., scale bar 100 *μ*m. (H) Gregarine gametocysts, scale bar 100 *μ*m. (I) Apostome *Hyalophysa lynni*, scale bar 40 *μ*m.

Parasites belonging to 10 genera of 6 classes were found to infect white shrimp in the Charleston Harbor watershed (Table 2; Fig. 2). Of the 597 white shrimp specimens examined (post-larvae to adult), 82% (491) were infected with at least 1 type of parasite. These parasites included 6 helminths [4 cestodes (1 lecanicephalidean, 1 cyclophyllidean and 2 trypanorhynchs), 1 plagiorchiid digenean and 1 rhabditid nematode], 2 ciliates (1 sessilid and 1 apostome), 1 gregarine and 1 microsporidian. All worms were found in a larval stage (i.e., metacercariae for the plagiorchiid, plerocerci for trypanorhynchs and juveniles for the rhabditid). Four of the parasite taxa were able to be identified to species-level using DNA sequencing (Table 2). Sequences from the nematode and 2 of the cestodes were only 96–97% similar to any sequence in GenBank and thus could only be identified to genus level (Table 2). Gregarine specimens could not be amplified or sequenced. Sequences from this study were deposited into GenBank under accession numbers OL456224–OL456226 and OL467311–OL467319 [platyhelminthes (*n* = 6), ciliate (*n* = 1), nematodes (*n* = 3) and microsporidians (*n* = 2)]. Parasites will hereafter be referenced as gregarines, apostomes, sessilids, microsporidians, rhabditids, cyclophyllids, lecanicephalideans, trypanorhynchs and plagiorchiids (Table 2).

### Ontogenetic and spatial variation in parasite communities

Prevalence and mean intensity or relative abundance of parasite infections varied across localities and white shrimp life stages (Table 3). Juvenile shrimp had significantly lower prevalence of infection by the rhabditid, lecanicephalidean and trypanorhynchs than adult shrimp (Table 4). Subadult shrimp had significantly higher prevalence of infection by the plagiorchiid and rhabditid than juvenile shrimp, but significantly lower prevalence of infection by the sessilid and lecanicephalidean than adult shrimp (Table 4). White shrimp collected at the open water Charleston Harbor localities had a higher prevalence of infection by the rhabditid, lecanicephalidean, trypanorhynchs and plagiorchiid, but lower prevalence of infection by the sessilid than those collected in the Ashley River and Wando River watersheds (the rhabditid was not collected from the Wando River; Tables 3 and 4). Parasite prevalence did not differ significantly between the Ashley River and Wando River watersheds (Tables 3 and 4).

**Table 3.** Prevalence (%)/mean intensity or relative abundance (±s.e.) of black gill and parasite infections by shrimp life stage and by locality

Parasite and black gill	Shrimp life stage				Locality		
	Post-larvae	Juveniles	Subadults	Adults	Wando River	Ashley River	Harbor
*N*	54	428	47	68	181	201	161
Gregarine	0/NA	59/NA	83/NA	82/NA	56/NA	57/NA	81/NA
Apostome^a^	0.0	43/0.6 (0.06)	64/2.8 (0.8)	59/1.0 (0.2)	34/0.4 (0.1)	45/0.4 (0.1)	62/1.8 (0.23)
Sessilid^a^	0.0	32/51.7 (2.6)	4/45.8 (7.9)	0/ NA	31/47.8 (3.8)	42/54.6 (3.7)	1/16.7 (1.3)
Microsporidian	0/NA	<1/NA	0/NA	0/NA	0/NA	0/NA	1/NA
Rhabditid	0.0	3/1.6 (0.08)	43/2.3 (0.4)	69/2.5 (0.3)	0/NA	1/1.00 (0.1)	50/2.3 (0.12)
Cyclophyllidean	NA	0/NA	4/NA	3/NA	0/NA	0/NA	3/NA
Lecanicephalidean	4/0.67	2/1.1 (0.05)	4/1.0 (0.1)	25/2.4 (0.5)	2/1.00 (0.1)	2/1.3 (0.1)	13/2.1 (0.3)
Trypanorhynch	0.0	47/5.6 (0.5)	83/14.9 (2.4)	90/13.1 (2.0)	36/1.8 (0.1)	49/6.1 (0.7)	86/12.9 (1.2)
Plagiorchiid	2/1	58/7.7 (0.9)	98/23.5 (3.4)	100/22.2 (2.5)	57/3.8 (0.3)	52/4.0 (0.5)	96/23.9 (2.1)
Black gill	0/NA	8/NA	51/NA	41/NA	1/NA	5/NA	50/NA

a See *Materials and methods: shrimp gill examination and ciliate abundance* for differences in abundance calculations for these 2 ciliate parasites.

**Table 4.** Pairwise *P* values from Tukey's *post hoc* tests of logistic regression models that examined parasite presence across shrimp life stages and localities (*n* = 532). Shrimp life stage and locality were significant factors (*P* < 0.01) in their respective models based on likelihood ratio tests unless indicated by ‘n.s.’ for not significant, in which case pairwise analyses were not conducted.

Parasite	Shrimp life stage			Locality		
	Juveniles *vs* subadults	Juveniles *vs* adults	Subadults *vs* adults	Wando River *vs* Ashley River	Wando River *vs* Harbor	Ashley River *vs* Harbor
Gregarine	0.173	0.235	0.939	0.995	0.102	0.113
Apostome	n.s.	n.s.	n.s.	n.s.	n.s.	n.s.
Sessilid	**<0**.**001**	**<0**.**001**	**<0**.**001**	0.210	**<0.001**	**<0**.**001**
Meiodihaplophasid	n.s.	n.s.	n.s.	n.s.	n.s.	n.s.
Rhabditid	**0**.**011**	**<0**.**001**	0.113	1	1	**<0**.**001**
Lecanicephalidean	0.724	**<0**.**001**	**0**.**038**	0.995	**0.043**	**0**.**034**
Trypanorhynch	0.145	**0**.**008**	0.574	0.138	**<0.001**	**<0**.**001**
Plagiorchiid	**0**.**012**	0.999	0.999	0.559	**<0.001**	**<0**.**001**

Significance at *P* < 0.05 of pairwise tests is indicated in bold font.

**Fig. 3.**
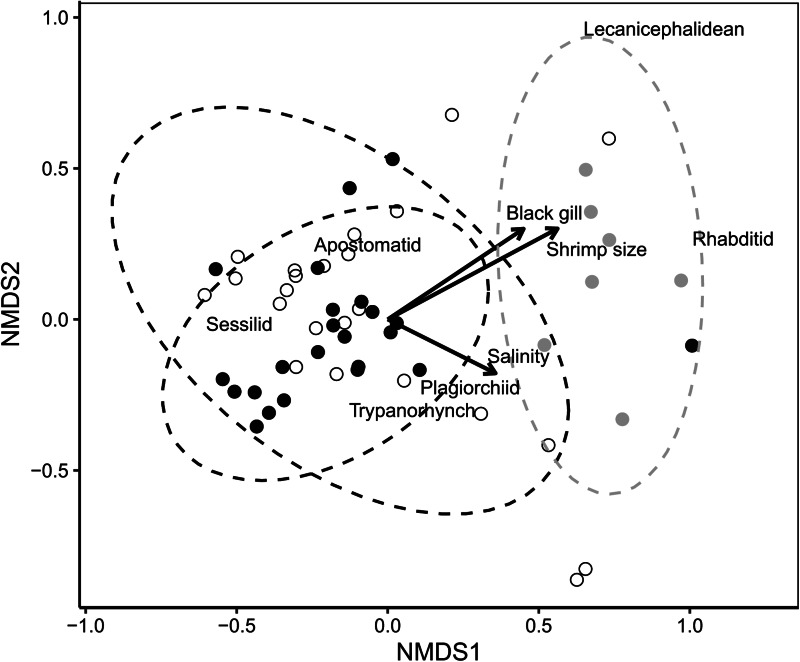
Non-metric multi-dimensional scaling (NMDS) plot of white shrimp infected with parasites in the greater Charleston Harbor watershed, South Carolina, USA (stress = 0.11). Data are presented by collection localities: Ashley River (filled black circles), Wando River (open circles) and Charleston Harbor (filled grey circles). Vectors represent significant (*P* ⩽ 0.01) factors related to community structure; taxa names (e.g. Sessilid) highlight locations in ordinal space most associated with those taxa. Dashed ovals represent data ellipses for parasite communities from each locality.

Parasite community structure varied significantly among both white shrimp life stages (PERMANOVA, *n* = 65, *R*^2^ = 0.24, *P* = 0.001) and localities (PERMANOVA, *n* = 52, *R*^2^ = 0.31, *P* = 0.001). The parasite communities of juveniles were significantly different from those of both adults and subadults (*P* = 0.002), which were not significantly different from each other (*P* = 0.233). Parasite community dissimilarities were also significantly correlated with black gill score (*R*^2^ = 0.29, *P* = 0.002), shrimp length (*R*^2^ = 0.41, *P* = 0.001), salinity (*R*^2^ = 0.16, *P* = 0.022), but not water temperature (*R*^2^ = 0.11, *P* = 0.068; Fig. 3). The apostome and the black gill score vectors were both located at higher *y*-axis values, but trypanorhynchs and plagiorchiids were located at lower *y*-axis values, highlighting that these factors were associated with different communities of parasites (Fig. 3).

Significant indicators of white shrimp collected at open water Charleston Harbor localities included the rhabditid (IV = 0.998, *P* = 0.001), plagiorchiid (IV = 0.907, *P* = 0.001), apostome (IV = 0.868, *P* = 0.001), trypanorhynch (IV = 0.855, *P* = 0.001) and lecanicephalidean (IV = 0.772, *P* = 0.001), while the lecanicephalidean (IV = 0.852, *P* = 0.005) and rhabditid (IV = 0.799, *P* = 0.005) were significant indicators of adult white shrimp. The sessilid was a significant indicator of juvenile white shrimp (IV = 0.355; *P* = 0.005) and of shrimp collected in the Ashley River watershed (IV = 0.767; *P* = 0.001).

### Parasite communities and black gill

**Fig. 4.**
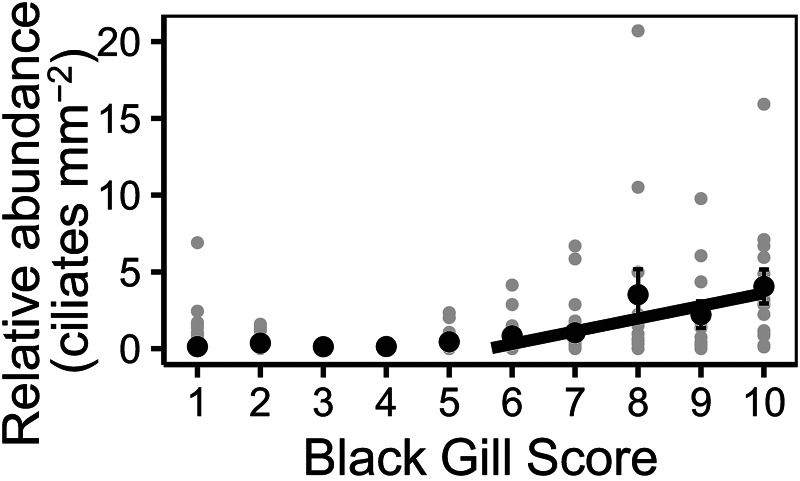
Black gill score related to apostome ciliate *Hyalophysa lynni* abundance for individual white shrimp (filled grey circles) and mean abundances (filled black circles ± s.e.; *n* = 306). Segmented regression showed a significant relationship between black gill score (when ⩾6) and apostome abundance on individual shrimp.

No post-larval white shrimp exhibited macroscopic evidence of black gill, which was almost exclusively found in specimens collected at open water Charleston Harbor localities, regardless of shrimp life stage. Black gill score was a significant vector associated with parasite community structure (Fig. 3). For shrimp collected in open water habitats in the fall, parasite communities between shrimp with and without black were significantly different (PERMANOVA, *n* = 9, *R*^2^ = 0.31, *P* = 0.008). Indicator species analyses showed that only the apostome was a significant indicator of shrimp with black gill (IV = 0.93; *P* = 0.005). The apostome was observed across 3 localities (Table 3). Juvenile, subadult and adult white shrimp were all observed with both black gill and apostome infections (Table 3), but significantly more adult white shrimp were infected with the apostome ciliate than exhibited black gill (binomial test *P* < 0.001). A positive slope existed between black gill scores ⩾6 (modeled breakpoint = 5.68 and the relative abundance of the apostome on the gills of white shrimp collected during the black gill season (slope = 0.83, confidence interval = 0.35 – 1.3), but there was no significant relationship between these 2 variables for scores of ⩽5 (slope = 0.025, confidence interval = −0.47 – 0.35, Fig. 4).

**Fig. 5.**
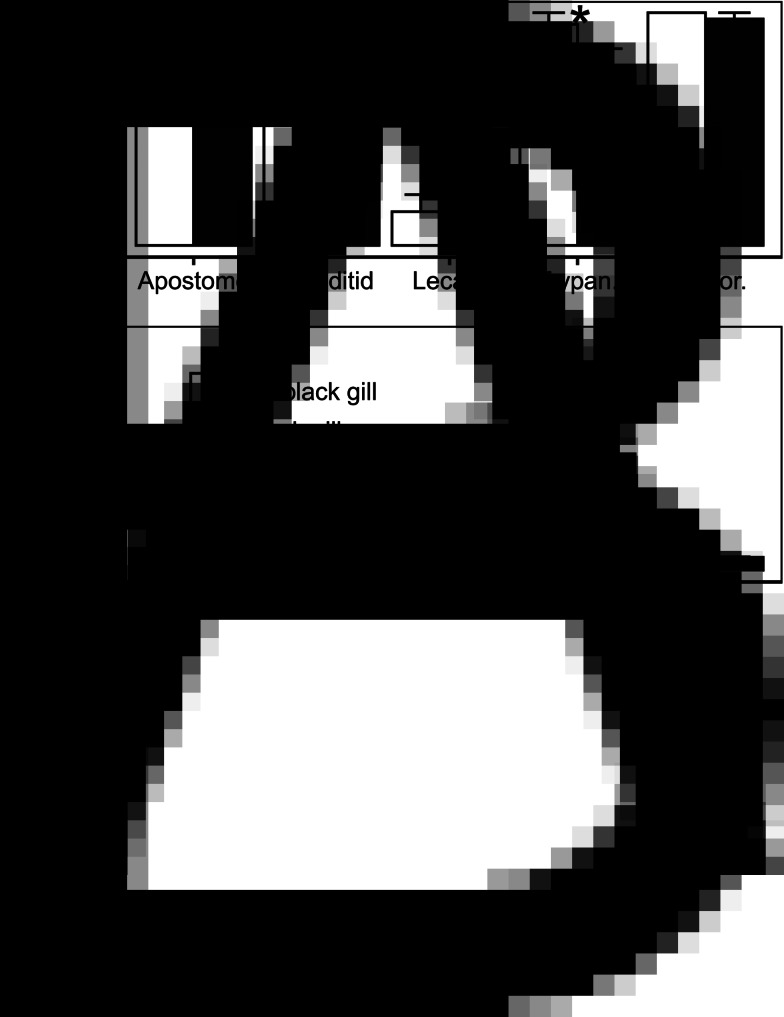
Infection metrics of parasites in white shrimp with black gill (black) and without black gill (white). Prevalence presented as (A) the percentage of shrimp infected and (B) mean intensity (±s.e.) and mean relative abundance (±s.e.) (for the apostome). Asterisks indicate significant differences between groups for prevalence (logistic regression, Table 5) and mean intensity or mean relative abundance (general linear mixed-effects model, Table 5).

Generalized mixed-effects models on prevalence showed parasite (*χ*^2^ = 275.9, *P* < 0.001) and black gill (*χ*^2^ = 24.8, *P* < 0.001) as significant factors, with individual shrimp (s.d. = 0.341) and collection month (s.d. = 0.211) included as random effects to account for sampling design. *Post hoc* analysis showed prevalence of infection by the trypanorhynchs, and with marginal significance of the rhabditid, was higher in shrimp without black gill than those with this condition (Table 5). The opposite occurred for the apostome, for which prevalence was higher in shrimp with black gill (Table 5; Fig. 5A). Generalized mixed-effects models on intensity showed parasite (*χ*^2^ = 20.8, *P* = 0.008) and black gill (*χ*^2^ = 22.1, *P* < 0.001) as significant factors, with individual shrimp (s.d. = 0.212) and collection month (s.d. = 0.001) included as random effects to account for sampling design. *Post hoc* analysis showed mean intensities of the rhabditid, and with marginal significance of the trypanorhynchs, were higher in shrimp without black gill than in those with this condition (Table 5; Fig. 5B). This pattern was again reversed for the apostome (Table 5; Fig. 5B). There was no significant association between the presence or intensity of the lecanicephalidean or plagiorchiid and black gill status.

**Table 5.** Marginal means pairwise contrasts from mixed-effects models of parasite prevalence or intensity in shrimp with and without black gill

Parasite	Prevalence		Intensity	
	Estimate	*P* value	Estimate	*P* value
Apostome	−1.767	**0**.**001**	−0.618	**0**.**001**
Rhabditid	0.738	0.057	0.416	**0**.**027**
Lecanicephalidean	−0.087	0.870	0.152	0.418
Trypanorhynch	1.443	**0**.**031**	0.350	0.062
Plagiorchiid	15.055	0.992	0.224	0.233

Significance at *P* < 0.05 is indicated with bold font.

## Discussion

The analyses presented here demonstrate differences in the parasite communities of white shrimp that were associated with localities (i.e., tidal creek *vs* open water habitats), host life stages and the occurrence of black gill. Juvenile white shrimp generally live in tidal creeks until they reach a certain size or level of maturity at which time they egress towards more saline waters in the summer and fall (Lindner and Cook, 1967). While the habitat and life stage of white shrimp were both significantly related to the composition of the parasite community in this study, the confounding nature of these variables makes it difficult to tease apart the individual effects of these factors. Despite this limitation, this study allowed for a deeper understanding of the ontogenetic and spatial dynamics of white shrimp parasite community composition, as well as the potential physiological impacts of parasites on this economically and ecologically important shrimp species.

To our knowledge, the only previous helminth survey of white shrimp in the Georgia Bight was limited to a total of 29 specimens and reported infection by ‘cestodes, trematodes and nematodes’ (Tripp and Turner, 1983). All parasites encountered herein have previously been reported from penaeid shrimp, including white shrimp in the Gulf of Mexico (Overstreet, 1973; Couch, 1978; Deardorff and Overstreet, 1981; Fontaine, 1985; Carreon *et al*., 2018), with the exception of the apostome *H. lynni*, which was only recently described from white shrimp from the South Atlantic Bight (Landers *et al*., 2020). An unidentified apostome ciliate on shrimp gills was associated with black gill in the Gulf of Mexico, albeit prior to the description of *H. lynni* (Overstreet, 1973; Couch, 1978).

In host–parasite systems, the diversity of parasites often changes ontogenetically with habitat and diet (Muñoz and Zamora, 2011; Münster *et al*., 2015). As such, higher prevalence and mean intensities of infection by the rhabditid, trypanorhynchs and lecanicephalidean in subadult and adult white shrimp are potentially attributable to the nature of the life cycles of these parasites. These 3 parasites infect white shrimp *via* their ingestion of either free-living stages or infected planktonic hosts (Johnson, 1975; Carreon *et al*., 2018). Thus, as shrimp age, opportunities for infection by these parasites increase and parasites can accumulate in their hosts.

The apostome ciliate was not found in post-larval white shrimp but it did infect all other life stages and was found across localities. Frischer *et al*. (2017) found correlations between the presence of this ciliate and black gill suggesting a linear response of the degree of gill melanization to the level of apostome infection. Results herein confirm such a relationship; however, it does not appear linear according to our analyses as many individuals infected with this ciliate did not exhibit black gill. Our data also indicate that a *H. lynni* abundance threshold may exist whereby only high abundances are associated with gill melanization (i.e., black gill). A delay between infection and melanization of the gill tissue, or the host response eliminating suitable parasite habitat leading to no infection at extreme levels of melanization (*sensu* Frischer *et al*., 2017) could also contribute to the non-linearity of this relationship.

Black gill is indicative of an immune response (Lightner and Redman, 1977; Martin *et al*., 2000; Cerenius *et al*., 2010) that can generate suboptimal living conditions for endoparasites (Burnett and Burnett, 2015) given its negative impact on the physiology of the hosts. Hence, activated immune response in shrimp with black gill could lead to the observed reduced prevalence and/or intensities of trypanorhynchs and rhabditids in white shrimp with black gill compared to those without black gill. Alternatively, given the role of the hepatopancreas in the crustacean immune system (Rőszer, 2014; Cao *et al*., 2021), damage caused by trypanorhynchs, which embed deeply in this organ and alter its function in portunid crabs (Gurney *et al*., 2006), could lead to immunosuppression. This could explain significantly higher prevalence of infection of white shrimp without black gill and immunosuppression could facilitate infection by other parasites, including the rhabditid that was also found in higher intensities in shrimp without black gill. Thus, while the apostome ciliate *H. lynni* remains a significant factor associated with black gill outbreaks and the degree of gill melanization in individual white shrimp, other parasites such as the trypanorhynchs and rhabditids may also influence the patterns of black gill. To further support the role of parasitic co-infection in black gill occurrence, analyses of fisheries-independent data show no significant relationship between black gill prevalence and white shrimp abundance (Kendrick *et al*., 2021). Environmental factors, such as salinity and temperature, also have the potential to influence both patterns of black gill (Fowler *et al*., 2018; Swinford and Anderson, 2021) as well as parasite communities (Koprivnikar *et al*., 2014; Strepparava *et al*., 2018), such that future studies should investigate the potential interrelatedness of biotic and abiotic factors in determining white shrimp health metrics.

## Data Availability

The data that support the findings of this study are available from the corresponding author, MRK, upon reasonable request.

## Supplementary material

For supplementary material accompanying this paper visit https://doi.org/10.1017/S0031182022001597. click here to view supplementary material

## References

[ref1] Altschul SF, Gish W, Miller W, Myers EW and Lipman DJ (1990) Basic local alignment search tool. Journal of Molecular Biology 215, 403–410. 2231712 10.1016/S0022-2836(05)80360-2

[ref2] Bates D, Maechler M, Bolker B and Walker S (2015) Fitting linear mixed-effects models using lme4. Journal of Statistical Software 67, 1–48.

[ref3] Budischak SA, Sakamoto K, Megow LC, Cummings KR, Urban Jr JF and Ezenwa VO (2015) Resource limitation alters the consequences of co-infection for both hosts and parasites. International Journal of Parasitology 45, 455–463. 25812832 10.1016/j.ijpara.2015.02.005

[ref4] Burnett KG and Burnett LE (2015) Respiratory and metabolic impacts of crustacean immunity: are there implications for the insects? Integrative and Comparative Biology 55, 856–868. 26223773 10.1093/icb/icv094

[ref5] Bush AO, Lafferty K, Lotz J and Shostak AW (1997) Parasitology meets ecology on its own terms: Margolis, *et al*. Revisited. Journal of Parasitology 83, 575–583. 9267395

[ref6] Byers JE (2021) Marine parasites and disease in the era of global climate change. Annual Review of Marine Science 13, 397–420. 10.1146/annurev-marine-031920-100429 32520636

[ref7] Cao X-T, Pan X-Y, Sun M, Liu Y and Lan J-F (2021) Hepatopancreas-specific lectin participates in the antibacterial immune response by regulating the expression of antibacterial proteins. Frontiers in Immunology 12, 679767. 34177924 10.3389/fimmu.2021.679767 PMC8226264

[ref8] Carreon N, Faulkes Z and Fredensborg BL (2018) *Polypocephalus* sp. infects the nervous system and increases activity of commercially harvested white shrimp (*Litopenaeus setiferus*). Journal of Parasitology 97, 755–759. 10.1645/GE-2749.1 21506800

[ref9] Cerenius L, Kawabata SI, Lee BL, Nonaka M and Soderhall K (2010) Proteolytic cascades and their involvement in invertebrate immunity. Trends in Biochemical Science 35, 575–583. 10.1016/j.tibs.2010.04.006 20541942

[ref10] Clay PA and Rudolf VH (2019) How parasite interaction strategies alter virulence evolution in multi-parasite communities. Evolution 73, 2189–2203. 31506940 10.1111/evo.13843

[ref11] Couch JA (1978) Diseases, parasites, and toxic responses of commercial penaeid shrimps of the Gulf of Mexico and South Atlantic coasts of North America. Fishery Bulletin 76, 1–44.

[ref12] Deardorff TL and Overstreet RM (1981) Larval *Hysterothylacium* (= *Thynnascaris*) (Nematoda: Anisakidae) from fishes and invertebrates in the Gulf of Mexico. Proceedings of the Helminthological Society of Washington 48, 113–126.

[ref13] De Cáceres M and Legendre P (2009) Associations between species and groups of sites: indices and statistical inference. Ecology 90, 3566–3574. 20120823 10.1890/08-1823.1

[ref14] DeLancey LB, Jenkins JE and Whitaker JD (1994) Results of long-term, seasonal sampling for *Penaeus* postlarvae at Breach Inlet, South Carolina. Fishery Bulletin 92, 633–640.

[ref15] Del Río-Rodríguez RE, Pech D, Soto-Rodriguez SA, Gomez-Solano MI and Sosa-Lopez A (2013) A ten-month diseases survey on wild *Litopenaeus setiferus* (Decapoda: Penaeidae) from southern Gulf of Mexico. Revista de Biologica Tropical 61, 1175–1188. 24027916

[ref16] Diakin A, Paskerova GG, Simdyanov TG, Aleoshin VV and Valigurová A (2016) Morphology and molecular phylogeny of coelomic gregarines (Apicomplexa) with different types of motility: *Urospora ovalis* and *U. travisiae* from the polychaete *Travisia forbesii*. Protist 167, 279–301. 27239726 10.1016/j.protis.2016.05.001

[ref17] Domínguez-Machín ME, Hernández-Vergara MP, Jiménez-García I, Simá-Álvarez R and Rodríguez-Canul R (2011) Survey of protozoan, helminth and viral infections in shrimp *Litopenaeus setiferus* and prawn *Macrobrachium acanthurus* native to the Jamapa River region, Mexico. Diseases of Aquatic Organisms 96, 97–103. 22013749 10.3354/dao02392

[ref18] Dufrene M and Legendre P (1997) Species assemblages and indicator species: the need for a flexible asymmetrical approach. Ecological Monographs 67, 345–367.

[ref19] Fontaine CT (1985) A survey of potential disease-causing organisms in bait shrimp from West Galveston Bay, Texas. NOAA Technical Memorandum NMFS-SEC 169, 1–63.

[ref20] Food and Agriculture Organization of the United Nations (FAO) (2018) The State of World Fisheries and Aquaculture 2018 – Meeting the Sustainable Development Goals. Rome: License, CC BY-NC-SA 3.0 IGO.

[ref21] Fowler AE, Leffler JW, Johnson SP, DeLancey LB and Sanger DM (2018) Relationships between meteorological and water quality variables and fisheries-independent white shrimp (*Litopenaeus setiferus*) catch in the ACE basin NERR, South Carolina. Estuaries and Coasts 41, 79–88.

[ref22] Frischer ME, Lee R, Price AR, Walters TL, Bassette MA, Verdiyev R, Torris MC, Bulski K, Geer PJ, Powell SA, Walker AN and Landers SC (2017) Causes, diagnostics, and distribution of an ongoing penaeid shrimp black gill epidemic in the U.S. South Atlantic Bight. Journal of Shellfish Research 36, 487–500.

[ref23] Frischer ME, Landers SC, Walker AN, Powell SA and Lee RF (2022) Black gill in marine decapod crustaceans: a review. Reviews in Fisheries Science & Aquaculture 30, 1–22.

[ref24] Fujiwara M, Zhou C, Acres C and Martinez-Andrade F (2016) Interaction between penaeid shrimp and fish populations in the Gulf of Mexico: importance of shrimp as forage species. PLoS ONE. doi: 10.1371/journal.pone.0166479 PMC5104333 27832213

[ref25] Ghate H (1984) Gill melanization and heavy metals in freshwater prawns. Indian Journal of Fisheries 31, 389–393.

[ref26] Ghosh K and Weiss LM (2009) Molecular diagnostic tests for Microsporidia. Interdisciplinary Perspectives on Infectious Diseases. doi: 10.1155/2009/926521 PMC2719812 19657457

[ref27] Gillet R (2008) Global study of shrimp fisheries. Part II. Shrimp fisheries in selected countries. Shrimp fishing in the United States of America. FAO Fisheries Technical Paper 475, pp. 289–312. FAO Fiji.

[ref28] Gleichsner AM, Reinhart K and Minchella DJ (2018) Of mice and worms: are co-infections with unrelated parasite strains more damaging to definitive hosts? International Journal of Parasitology 48, 881–885. 30059691 10.1016/j.ijpara.2018.05.004

[ref29] Graham AL (2008) Ecological rules governing helminth-microparasite coinfection. Proceedings of the National Academy of Sciences of the USA 105, 566–570. 18182496 10.1073/pnas.0707221105 PMC2206576

[ref30] Guo ZL, Liu S, Hu SM, Li T, Huang YS, Liu GX, Zhang H and Lin SJ (2012) Prevalent ciliate symbiosis on copepods: high genetic diversity and wide distribution detected using small subunit ribosomal RNA gene. PLoS ONE. doi: 10.1371/journal.pone.0044847 PMC3443111 23024768

[ref31] Gurney RH, Johnston DJ and Nowak BF (2006) The effect of parasitism by trypanorhynch plerocercoids (Cestoda, Trypanorhyncha) on the digestive enzyme activity of *Carcinus maenas* (Linnaeus, 1758) (Decapoda, Portunidae). Crustaceana 79, 663–675.

[ref32] Hellard E, Fouchet D, Vavre F and Pontier D (2015) Parasite-parasite interactions in the wild: how to detect them? Trends in Parasitology 31, 640–652. 26440785 10.1016/j.pt.2015.07.005

[ref33] Hervé M (2021) RVAideMemoire: testing and plotting procedures for biostatistics. R package version 0.9–79. Available at https://CRAN.R-project.org/package=RVAideMemoire.

[ref34] Hollebone AL (2006) An invasive crab in the South Atlantic Bight: friend or foe? (Thesis), 114 pp. Georgia Institute of Technology.

[ref35] Hothorn T, Bretz F and Westfall P (2008) Simultaneous inference in general parametric models. Biometrical Journal 50, 346–363. 18481363 10.1002/bimj.200810425

[ref36] Jensen K and Bullard SA (2010) Characterization of a diversity of tetraphyllidean and rhinebothriidean cestode larval types, with comments on host associations and life-cycles. International Journal of Parasitology 40, 889–910. 20026125 10.1016/j.ijpara.2009.11.015

[ref37] Johnson SK (1975) Handbook of shrimp diseases. Aquaculture 601, 1–27.

[ref38] Johnson WS and Allen DM (2005) Zooplankton of the Atlantic and Gulf Coasts: A Guide to Their Identification and Ecology. Baltimore, MD: Johns Hopkins University Press.

[ref39] Johnson NG, Burnett LE and Burnett KG (2011) Characterization of bacteria that trigger hemocytopenia in the Atlantic blue crab, *Callinectes sapidus*. Biological Bulletin 221, 164–175. 22042435 10.1086/BBLv221n2p164

[ref40] Karthikeyan V, Selvakumar A and Gopalakrishnan A (2015) A novel report of fungal pathogen *Aspergillus awamori* causing black gill infection on *Litopenaeus vannamei* (Pacific white shrimp). Aquaculture 444, 36–40.

[ref41] Kendrick MR, Brunson JF, Frischer ME and Kingsley-Smith PR (2021) Climate indices predict black gill prevalence in white shrimp *Penaeus setiferus* (Linnaeus, 1767) in South Carolina and Georgia, USA. Journal of Shellfish Research 40, 145–151.

[ref42] Koprivnikar J, Ellis D, Shim KC and Forbes MR (2014) Effects of temperature and salinity on emergence of *Gynaecotyla adunca* cercariae from the intertidal gastropod *Ilyanassa obsoleta*. Journal of Parasitology 100, 242–245. 24294898 10.1645/13-331.1

[ref43] Lafferty KD (2017) Marine infectious disease ecology. Annual Review of Ecology, Evolution, and Systematics 48, 473–496.

[ref44] Landers SC, Lee RF, Walters TL, Walker AN, Powell SA, Patel M and Frischer ME (2020) *Hyalophysa lynni* n. sp. (Ciliophora, Apostomatida), a new pathogenic ciliate and causative agent of shrimp black gill in penaeid shrimp. European Journal of Protistology 73, 1–10. 10.1016/j.ejop.2020.125673 32007803

[ref45] Leander BS, Clopton RE and Keeling PJ (2003) Phylogeny of gregarines (Apicomplexa) as inferred from small-subunit rDNA and *β*-tubulin. International Journal of Systematic and Evolutionary Microbiology 53, 345–354. 12656194 10.1099/ijs.0.02284-0

[ref46] Lefèvre T, Lebarbenchon C, Gauthier-Clerc M, Missé D, Poulin R and Thomas F (2008) The ecological significance of manipulative parasites. Trends in Ecology and Evolution 24, 41–48. 19026461 10.1016/j.tree.2008.08.007

[ref47] Lenth RV (2022) emmeans: Estimated marginal means, aka least-squares means. R package version 1.7.2. Available at https://CRAN.R-project.org/package=emmeans.

[ref48] Lightner DV and Redman R (1977) Histochemical demonstration of melanin in cellular inflammatory processes of penaeid shrimp. Journal of Invertebrate Pathology 30, 298–302.

[ref49] Lindner MJ and Cook HL (1967) Synopsis of biological data on the white shrimp *Penaeus setiferus*. Food and Agriculture Organization of the United Nations (FAO) Fisheries Reports 57, 1439–1469.

[ref50] Marcogliese DJ (2004) Parasites: small players with crucial roles in the ecological theater. EcoHealth 1, 151–164.

[ref51] Martin GG, Quintero M, Quigley M and Khosrovian H (2000) Elimination of sequestered material from the gills of decapod crustaceans. Journal of Crustacean Biology 20, 209–217.

[ref52] Muncy RJ (1984) Species profiles: life histories and environmental requirements of coastal fishes and invertebrates (Gulf of Mexico). *U.S. Fish and Wildlife Service Biological Report* 82/11.20.

[ref53] Muñoz G and Zamora L (2011) Ontogenetic variation in parasite infracommunities of the clingfish *Sicyases sanguineus* (Pisces: Gobiesocidae). Journal of Parasitology 97, 14–19. 21348601 10.1645/GE-2445.1

[ref54] Münster J, Klimpel S, Fock HO, MacKenzie K and Kuhn T (2015) Parasites as biological tags to track an ontogenetic shift in the feeding behavior of *Gadus morhua* off West and East Greenland. Parasitology Research 114, 2723–2733. 25899328 10.1007/s00436-015-4479-y

[ref55] Nadler SA and Hudspeth DSS (2000) Phylogeny of the Ascaridoidea (Nematoda: Ascaridida) based on three genes and morphology: hypotheses of structural and sequence evolution. Journal of Parasitology 86, 380–393. 10780561 10.1645/0022-3395(2000)086[0380:POTANA]2.0.CO;2

[ref56] National Marine Fisheries Service (NMFS) (2020) Fisheries of the United States, 2018. U.S. Department of Commerce, NOAA Current Fishery Statistics No. 2018 Available at https://www.fisheries.noaa.gov/national/commercial-fishing/fisheries-united-states-2018.

[ref57] Oksanen J, Blanchet F, Friendly M, Kindt R, Legendre P, McGlinn D, Minchin PR, O'Hara RB, Simpson GL, Solymos P, Stevens M, Henry H, Szoecs E and Wagner H (2019) Vegan: community ecology package. R package version 2.5–5. Available at https://CRAN.R-project.org/package=vegan.

[ref58] Overstreet RM (1973) Parasites of some penaeid shrimps with emphasis on reared hosts. Aquaculture 2, 105–140.

[ref59] Overstreet RM (1978) Marine Maladies? Worms, Germs, and Other Symbionts From the Northern Gulf of Mexico. Ocean Springs, Mississippi: Blossman Printing.

[ref60] Palm HW (2004) The Trypanorhyncha Diesing, 1863. Bogor, Indonesia: PKSPL-IPB Press.

[ref61] Pedersen AB and Fenton A (2007) Emphasizing the ecology in parasite community ecology. Trends in Ecology and Evolution 22, 133–139. 17137676 10.1016/j.tree.2006.11.005

[ref62] R Core Team (2019) R: A Language and Environment for Statistical Computing. Vienna, Austria: R Foundation for Statistical Computing.

[ref63] Reckardt K and Kerth G (2009) Does the mode of transmission between hosts affect the host choice strategies of parasites? Implications from a field study on bat fly and wing mite infestation of Bechstein's bats. Oikos 118, 183–190.

[ref64] Rodríguez ML, Terrazas I, Márquez R and Bojalil R (1999) Susceptibility to *Trypanosoma cruzi* is modified by a previous non-related infection. Parasite Immunology 21, 177–185. 10320615 10.1046/j.1365-3024.1999.00218.x

[ref65] Rőszer T (2014) The invertebrate midintestinal gland (‘hepatopancreas’) is an evolutionary forerunner in the integration of immunity and metabolism. Cell Tissue Research 358, 685–695. 25174684 10.1007/s00441-014-1985-7

[ref66] Rueckart S and Leander BS (2009) Molecular phylogeny and surface morphology of marine archigregarines (Apicomplexa), *Selenidium* spp., *Filipodium phascolosomae* n. sp., and *Platyproteum* n. g. and comb. from North-Eastern Pacific peanut worms (Sipuncula). Journal of Eukaryotic Microbiology 56, 428–439. 19737195 10.1111/j.1550-7408.2009.00422.x

[ref67] Scharf FS and Schlight KK (2000) Feeding habits of red drum (*Sciaenops ocellatus*) in Galveston Bay, Texas: seasonal diet variation and predator-prey size relationships. Estuaries 23, 128–139.

[ref68] Shearman RK and Lentz SJ (2010) Long-term sea surface temperature variability along the U.S. east coast. Journal of Physical Oceanography 40, 1004–1017.

[ref69] Sokolova Y, Pelin A, Hawke J and Corradi N (2015) Morphology and phylogeny of *Agmasoma penaei* (Microsporidia) from the type host, *Litopenaeus setiferus*, and the type locality, Louisiana, USA. International Journal of Parasitology 45, 1–16. 25449947 10.1016/j.ijpara.2014.07.013

[ref70] Strepparava N, Segner H, Ros A, Hartikainen H, Schmidt-Posthaus H and Wahli T (2018) Temperature-related parasite infection dynamics: the case of proliferative kidney disease of brown trout. Parasitology 145, 281–291. 28831940 10.1017/S0031182017001482

[ref71] Swinford JL and Anderson JD (2021) Prevalence of black gill (*Hyalophysa lynni*) in white shrimp *Litopenaeus setiferus* and brown shrimp *Farfantepenaeus aztecus* along the Texas Gulf Coast. Marine and Coastal Fisheries 13, 263–274.

[ref72] Tripp MR and Turner RM (1983) Helminth infections of some invertebrates of the Georgia Bight. Journal of Invertebrate Pathology 41, 57–67.

[ref73] Tuckey TD, Swinford JL, Fabrizio MC, Small HJ and Shields JD (2021) Penaeid shrimp in Chesapeake Bay: population growth and black gill disease syndrome. Marine and Coastal Fisheries 13, 159–173.

[ref74] Vaseeharan B and Ramasamy P (2003) Abundance of potentially pathogenic micro-organisms in *Penaeus monodon* larvae rearing systems in India. Microbiological Research 158, 299–308. 14717450 10.1078/0944-5013-00208

[ref75] Wenner EL, Knott DM, Barans CA, Wilde S, Blanton JO and Amft J (2005) Key factors influencing transport of white shrimp (*Litopenaeus setiferus*) post-larvae into the Ossabaw Sound system, Georgia, USA. Fisheries Oceanography 14, 175–194.

[ref76] Whitaker JD and Kingsley-Smith PR (2014) Sea science: an information/education series from the Marine Resources Division. Shrimp life cycle. Available at https://www.dnr.sc.gov/marine/pub/seascience/shrimpcycle.html (Accessed January 2020).

