# Supplementary material for: Ontogenetic and spatial variability in parasite communities of white shrimp Penaeus setiferus (Decapoda: Penaeidae)
Source: Parasitology. 2022 Dec 12;150(3):230–9. doi: 10.1017/S0031182022001597 (PMC10090638; doi:10.1017/S0031182022001597)
Supplement: Supplementary file 1 [file S0031182022001597sup001.docx]

**Supplementary material**

**Table S1.** Presence (+) and absence (-) of nine types of parasites within microhabitats of the shrimp. The shrimp hepatopancreas is denoted by HP and the shrimp nerve cord is denoted by NC.

|  | **Gills** | **HP** | **Anterior Cecum** | **Gut Lining** | **Rectum** | **Muscle Tissue** | **NC** | **Ovaries** | **Others*** |
| --- | --- | --- | --- | --- | --- | --- | --- | --- | --- |
|  |  |  |  |  |  |  |  |  |  |
| **Apostome Ciliates** | + | - | - | - | - | - | - | - | - |
| **Sessilid Ciliates** | + | - | - | - | - | - | - | - | - |
| **Gregarines** | - | - | - | - | + | - | - | - | - |
| **Meiodihaplophasids** | - | + | - | - | - | + | - | + | - |
| **Plagiorchiids** | + | + | - | + | ˗ | + | + | - | + |
| **Cyclophyllids** | - | - | - | + | - | - | - | - | - |
| **Lecanicephalideans** | - | - | - | - | - | - | + | - | - |
| **Rhabditids** | - | - | + | - | - | - | - | - | - |
| **Trypanorhynchs** | - | + | - | + | - | - | - | - | - |
|  |  |  |  |  |  |  |  |  |  |

*Others = Palps and Lining of the Heart
